# Genome-Wide Analysis of Tandem Repeats in Plants and Green Algae

**DOI:** 10.1534/g3.113.008524

**Published:** 2013-11-05

**Authors:** Zhixin Zhao, Cheng Guo, Sreeskandarajan Sutharzan, Pei Li, Craig S. Echt, Jie Zhang, Chun Liang

**Affiliations:** * Department of Botany, Miami University, Oxford, Ohio 45056; † Department of Automation, Xiamen University, Xiamen, 361005, China; ‡ Southern Research Station, USDA Forest Service, Saucier, Mississippi 39574; § State Key Laboratory for Biology of Plant Diseases and Insect Pests, Institute of Plant Protection, Chinese Academy of Agricultural Science, Beijing 100193, China

**Keywords:** tandem repeats, SSR, genomes, plants, green algae

## Abstract

Tandem repeats (TRs) extensively exist in the genomes of prokaryotes and eukaryotes. Based on the sequenced genomes and gene annotations of 31 plant and algal species in Phytozome version 8.0 (http://www.phytozome.net/), we examined TRs in a genome-wide scale, characterized their distributions and motif features, and explored their putative biological functions. Among the 31 species, no significant correlation was detected between the TR density and genome size. Interestingly, green alga *Chlamydomonas reinhardtii* (42,059 bp/Mbp) and castor bean *Ricinus communis* (55,454 bp/Mbp) showed much higher TR densities than all other species (13,209 bp/Mbp on average). In the 29 land plants, including 22 dicots, 5 monocots, and 2 bryophytes, 5′-UTR and upstream intergenic 200-nt (UI200) regions had the first and second highest TR densities, whereas in the two green algae (*C. reinhardtii* and *Volvox carteri*) the first and second highest densities were found in intron and coding sequence (CDS) regions, respectively. In CDS regions, trinucleotide and hexanucleotide motifs were those most frequently represented in all species. In intron regions, especially in the two green algae, significantly more TRs were detected near the intron–exon junctions. Within intergenic regions in dicots and monocots, more TRs were found near both the 5′ and 3′ ends of genes. GO annotation in two green algae revealed that the genes with TRs in introns are significantly involved in transcriptional and translational processing. As the first systematic examination of TRs in plant and green algal genomes, our study showed that TRs displayed nonrandom distribution for both intragenic and intergenic regions, suggesting that they have potential roles in transcriptional or translational regulation in plants and green algae.

Tandem repeats (TRs) are DNA sequence motifs that contain at least two adjacent repeating units. They extensively exist in prokaryotes and eukaryotes (Tautz and Renz 1984; Tóth *et al.* 2000; Sharma *et al.* 2007; Sureshkumar *et al.* 2009; Christians and Watt 2009; Orsi *et al.* 2010; Roorkiwal and Sharma 2011). Generally, two categories are given to distinguish TRs based on different repeat unit size: microsatellites [unit size: 1–6 or 1–10 bp; also known as simple sequence repeats (SSR)] and minisatellites (unit size: 10–60 or 10–100 bp) (Mayer *et al.* 2010; Gemayel *et al.* 2012). In plants and animals, SSRs are widely detected in both mRNAs (cDNA/ESTs) and genomes (Tautz and Renz 1984; Jurka and Pethiyagoda 1995; Tóth *et al.* 2000; Subramanian *et al.* 2003; Fujimori *et al.* 2003; Sharma *et al.* 2007; Gemayel *et al.* 2010). For example, through investigating SSRs (repeat unit size: 1–6 bp) using EST databases in 11 plant and green algal species, Victoria *et al.* (2011) found that dimer motifs have higher frequencies in green algae, bryophytes, and ferns, whereas trimer motifs are more frequent in flowering plants. Different from nuclear genomes, mitochondrial genomes appear to prefer mononucleotide repeats (A/T) first and dinucleotide repeats (AT) next in 16 investigated plant species (Kuntal and Sharma 2011). Although most research articles focus on SSRs, Mayer *et al.* (2010) found that in coding regions densities of longer TRs (unit size: 7–50 bp) in arthropoda *Daphnia pulex* are much higher than shorter TRs (unit size: 1–6 bp) and suggest the importance of including longer TRs in comparative analyses.

TRs are extremely mutable, with mutation rates that are much higher than other parts of the genome (Gemayel *et al.* 2010). Most mutations in TRs are caused by the changes in the number of the repeating units, not by point mutations (Verstrepen *et al.* 2005; Gemayel *et al.* 2010, 2012). In humans, such repeat number variants are related to some serious diseases or defects, such as fragile X syndrome (Verkerk *et al.* 1991), spinobulbar muscular atrophy (La Spada *et al.* 1991), and Huntington disease (Walker 2007). In plants, the well-known Bur-0 *IIL1* defect in *Arabidopsis thaliana* that generates a detrimental phenotype is caused by the expansion of triplet TTC/GAA in the intron of *IIL1* gene (Sureshkumar *et al.* 2009).

Through investigating TR density variation in a few plant and animal species, it has been concluded that there is no significant relationship between genome size and TR density in plants and animals (da Maia *et al.* 2009; Mayer *et al.* 2010). Based on EST data from two green algae, two mosses, a fern, a fern palm, the ginkgo tree, two conifers, 10 dicots, and five monocots, SSRs are found to have highly variable abundance among different species (von Stackelberg *et al.* 2006). Recently, a comparative analysis for 282 species including plants and animals shows no sequence conservation in centromere TRs (Melters *et al.* 2013). Moreover, TRs show a nonrandom distribution in many genomes and are often located within genes and regulatory regions (Streelman and Kocher 2002; Rockman and Wray 2002; Li *et al.* 2002; Martin *et al.* 2005; Legendre *et al.* 2007; Vinces *et al.* 2009). Variable TRs are abundant in genes that are involved in transcriptional regulation and morphogenesis in humans (Legendre *et al.* 2007). The 5′-UTRs have higher TR density among different genic regions in plants (Morgante *et al.* 2002; Fujimori *et al.* 2003; Zhang *et al.* 2006). In *A. thaliana*, for example, 5′-UTRs have the highest TR density and the abundant motifs are dinucleotide CT/GA and trinucleotide CTT/GAA (Zhang *et al.* 2006). In the yeast *Saccharomyces cerevisiae*, ∼25% genes possess TRs in their promoters, and the variations of repeat unit number can cause changes in gene expression and local nucleosome positioning (Vinces *et al.* 2009). Among coding sequences (CDS), the dominant repeat unit sizes are three-fold nucleotides (*e.g.*, trinucleotides and hexanucleotides) because it is assumed that such motifs are selected to avoid frame shift mutations that would affect translation (Legendre *et al.* 2007; Metzgar *et al.* 2002). In 42 fully sequenced prokaryotic genomes, the TR distributions in CDS are biased toward CDS termini, yielding U-shape TR density curves across the span of the CDS (Lin and Kussell 2011).

So far, no systematic research regarding TR variation and characterization has been conducted on a genome-wide scale in plants. The rapid advance of sequencing technologies has made a number of plant and algal genomes available to investigate the characteristics and distributions of TRs in both intragenic (*i.e.*, 5′-UTR, CDS, intron, and 3′-UTR) and intergenic regions. Using genome sequence data from 31 species (*i.e.*, 29 land plants and 2 green algae) released in Phytozome version 8.0 (http://www.phytozome.net/), we detected and characterized TRs and examined their distributions and variations in intragenic and intergenic regions. This research will facilitate our understanding of TRs and their potential biological functions in transcription or translation in land plants and green algae.

## Materials and Methods

### Collecting genomes and annotation data

**Figure 1:**
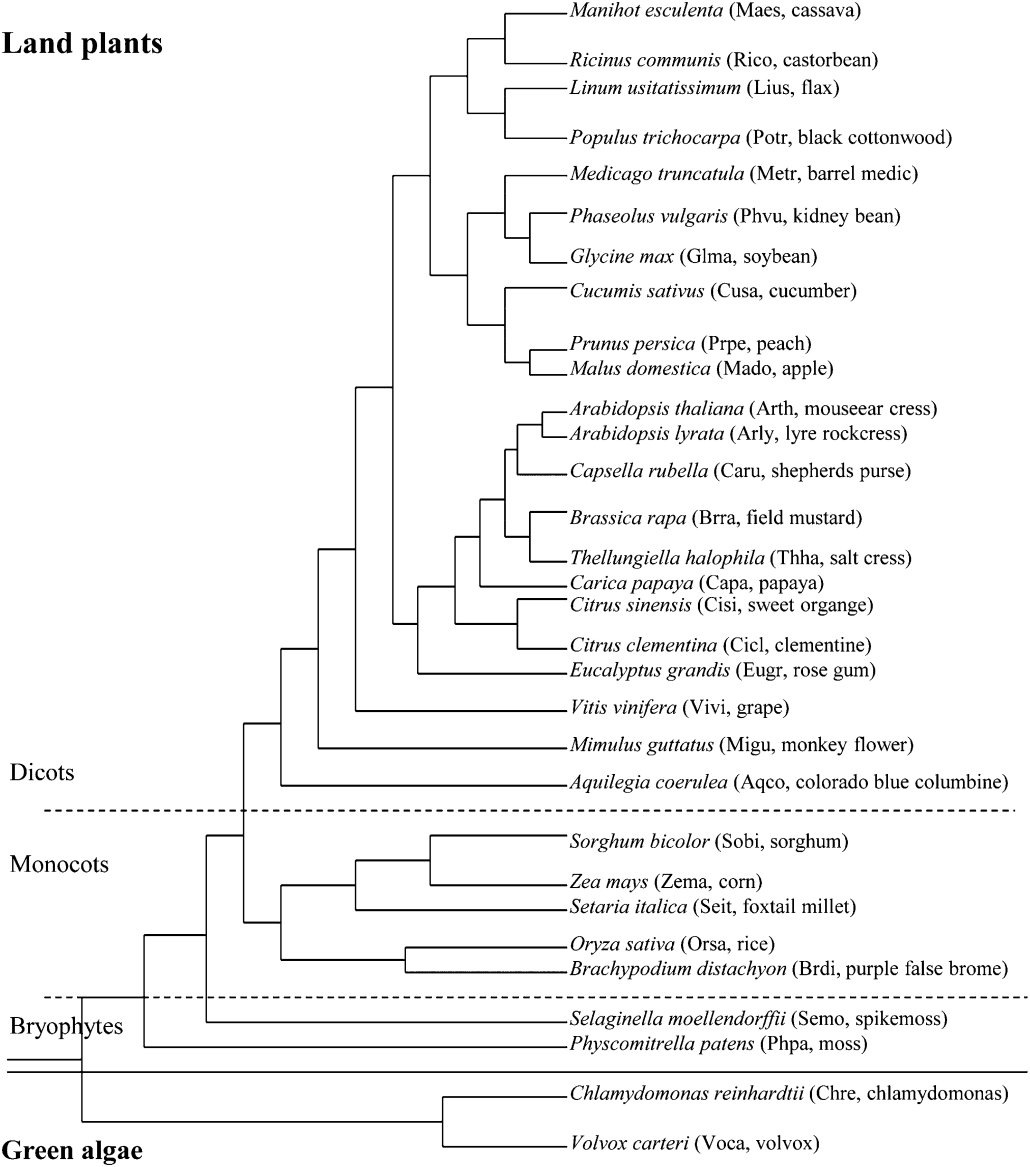
The phylogenic tree of 31 species showed in Phytozome version 8.0 (http://www.phytozome.net). The abbreviated names (the first two letters from both genus and species name are combined) and common names are listed in parentheses.

**Figure 2:**
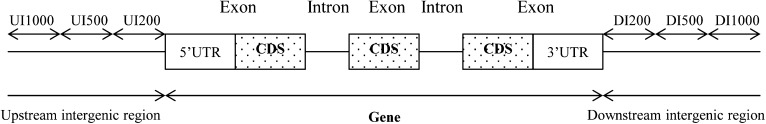
The schematic intragenic and intergenic regions used for TR analysis. UI200: 1–200 nt upstream of 5′UTR; UI500: 201–700 nt upstream of 5′UTR; UI1000: 701–1700 nt upstream of 5′UTR; DI200: 1–200 nt downstream of 3′UTR; DI500: 201–700 nt downstream of 3′UTR; and DI1000: 701–1700 nt downstream of 3′UTR.

The assembled genome sequences (including chromosomes, mitochondria, and chloroplasts) and gene annotations of the 31 species were downloaded from Phytozome version 8.0 (http://www.phytozome.net) (Figure 1 for the species list). Only valid nucleotides (A, T, G, and C) were counted when analyzing the sequences. For each species, the nucleotide sequences from whole genome were used for genome-wide TR detection and density calculation. According to the data extraction schema shown in Figure 2, individual intergenic and intragenic regions were also extracted and used for TR analysis. In Phytozome version 8.0, UTR annotations, including 5′-UTRs and 3′-UTRs, were not available for *Carica papaya*, *Brassica rapa*, *Linum usitatissimum*, and *Malus domestica*. Therefore, the UTR regions were not examined individually for these four species. However, the upstream and downstream intergenic regions (*e.g.*, UI1000, DI1000) were still examined based on the relevant gene start and end positions annotated for these four species (Figure 2). Perl (Practical Extraction and Report Language) was used to write codes to extract sequences, initiate TR detection, and parse results for downstream data analysis.

### TR detection and analysis

For both perfect and imperfect TR detection, we utilized a tandem repeat search tool for complete genomes (Phobos version 3.3.12) (Mayer *et al.* 2010). Considering the computational resource and execution time required for processing all 31 genomes, we adopted 1–50 bp as the repeat unit size, similar to what has been utilized previously by Mayer *et al.* (2010). The minimum length of the detected repeats needed to be at least 12 nt, and the minimum repeat alignment score for imperfect repeats was set as 12. As for the recursive TRs, only one motif was selected based on alphabetical ordering to be representative (Jurka and Pethiyagoda 1995). For example, AAG, AGA, and GAA were the repeat units of (AAG)n, but only AAG was selected to represent the repeat motif. Moreover, the TR motifs and their corresponding reverse complement motifs (*e.g.*, AAG and CTT motifs) were investigated separately. This was because genes are annotated in different strands (*i.e.*, + *vs.* −), there are plenty of sense and anti-sense transcripts reported recently for many genes (Gu *et al.* 2009; Kerin *et al.* 2012), emphasizing the importance of gene orientation in genome annotations, and a similar strategy had been adopted by others (Zhang *et al.* 2006; Kuntal and Sharma 2011).

TR density was defined by base pairs per megabase pairs (bp/Mbp), namely the length of detected TRs out of the total length of the sequences for detection. To enable comparison among different species or different regions (*e.g.*, intragenic *vs.* intergenic regions) within the same species, we normalized the TR densities and computed the relative density: for each species, the whole genome density was defined as 100, and then the relative density for a specific region was computed by the following: (TR density for a given region)/(the whole genome density). To investigate the TR distribution profiles within a given region, the sequence length of a specific region was first normalized to a 0–99 scale that contained 10 intervals of the same size (*e.g.*, 0–9, 10–19, …, 90–99). The motif percentages were then calculated for the 10 intervals based on their occurrences. In this way, the same intergenic or intragenic regions with different sequence lengths can be compared.

The 198 experimentally verified plant promoter sequences, which were extracted from −499 to 100 around the transcription start site (0 position in the coordinate), were downloaded from EPD (Eukaryotic Promoter Database; http://epd.vital-it.ch/seq_download.php#) (Périer *et al.* 2000). These promoter sequences were also scanned for perfect and imperfect TRs.

Based on *Chlamydomonas reinhardtii* GO annotation (version 4.0) from JGI (http://genome.jgi-psf.org/Chlre4/Chlre4.download.ftp.html), GOEAST (Gene Ontology Enrichment Analysis Software Toolkit)(Zheng and Wang 2008) was used to detect the significance of GO terms for the genes with TRs in introns. GO annotations in *Volvox carteri* were analyzed by annot8r (Schmid and Blaxter 2008) and ranked based on E-value. Pearson correlation (*r*) test statistics were conducted using Minitab 16 (www.minitab.com). The figures in the box plot were drawn using R (http://www.r-project.org/). Also through R, both ANOVA F-test and Tukey honestly significant difference (HSD) test (Yandell 1997) were performed for significance tests.

## Results

### The TR density variation among different genome sizes

**Figure 3:**
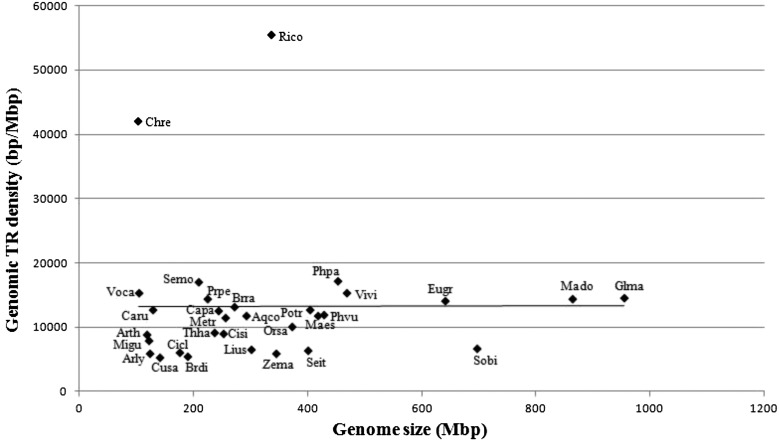
Genome size *vs.* genomic TR density in 31 land plants and green algae. The abbreviation name is based on the first two letters from both genus and species name (see Figure 1). The linear trendline was made using Excel.

The species that we examined span a large evolutionary distance, including two green algae, two mosses, five monocots, and 22 dicots (Figure 1). As shown in Figure 3, there was no correlation between genome sizes and TR densities (*r* = 0.010; *P* = 0.957). The mean TR density at the whole-genome level was 13,209 bp/Mbp (SD = 10,309) among all tested species, except in *C. reinhardtii* (42,059 bp/Mbp) and *Ricinus communis* (55,454 bp/Mbp), which showed dramatically higher TR densities than the other species. Excluding these two outliers (*C. reinhardtii* and *R. communis*), we still cannot find a significant correlation between genome sizes and TR densities among the remaining species (*r* = 0.311; *P* = 0.101) (Figure 3).

### The TR density variation in intragenic and intergenic regions

Sequences from functionally different intragenic regions (*i.e.*, 5′-UTR, CDS, intron and 3′-UTR) and progressively flanking upstream (*i.e.*, UI200, UI500, UI1000) and downstream (*i.e.*, DI200, DI500, and DI1000) intergenic regions were analyzed for TRs (Figure 2). UTR annotations were not available from Phytozome version 8.0 for four species (*C. papaya*, *B. rapa*, *L. usitatissimum*, and *M. domestica*); therefore, 5′-UTR and 3′-UTR were analyzed only for the remaining 27 species.

**Figure 4:**
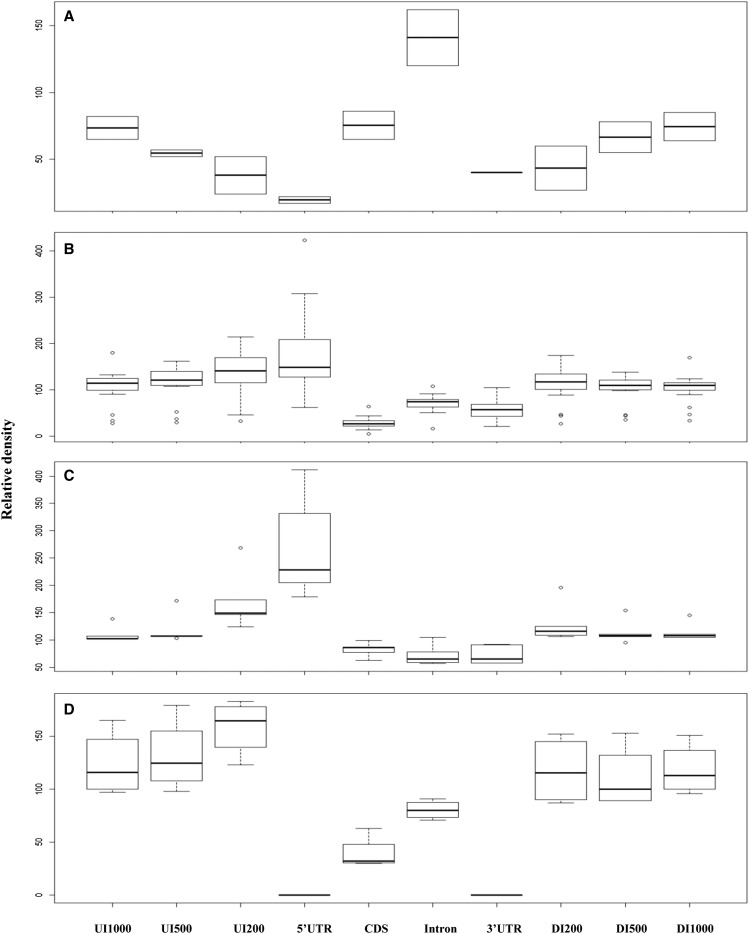
The relative TR densities in different intragenic and intergenic regions. (A) Two green algae. (B) Twenty species including dicots and bryophytes. (C) Five monocot land plants. (D) Four land plant species without UTR annotations.

We found that TRs showed clearly localization preferences among different intragenic and intergenic regions. In the two green algae, *C. reinhardtii* and *V. carteri* (Figure 4A and Supporting Information, Table S1, and Table S2), intron regions have the highest relative TR densities of 162 and 120, respectively, which are 1.62-times and 1.20-times of the relevant whole-genome TR densities (the whole-genome relative TR density is defined as 100 for each species). Based on F-test from ANOVA, the null hypothesis that all tested intergenic and intragenic regions have the equal mean relative TR densities can be rejected (*P* < 0.001), and Tukey HSD test (Yandell 1997) also showed a significant difference between the intron and each of the other regions (*P* < 0.05). In contrast, CDS regions had the second highest relative TR densities in the genic regions (86 and 65), whereas 5′-UTRs had the lowest (17 and 22). Interestingly, TRs in intergenic regions increased their relative densities away from the genes in these two green algae (Figure 4A, Table S1, and Table S2).

In the 29 land plants we examined, 5′-UTRs had the most significant and highest relative densities (*P* < 0.001, F-test from ANOVA; *P* < 0.01, HSD test) (Figure 4, B and C, Table S1, and Table S2) among different intragenic and intergenic regions. In the dicots and bryophytes, CDS regions had the significantly lowest relative TR densities (*P* < 0.01, F-test from ANOVA; *P* < 0.03, HSD test) (Figure 4B and Table S1) among different regions. In monocots, the relative densities of CDS, intron, and 3′-UTRs are similarly low (Figure 4 C, Table S1, and Table S2). Different from the two green algae, the intergenic regions in land plants generally show higher TR densities than their genomes average (Figure 4, B, C, and D, Table S1, and Table S2). Comparing all intergenic regions (Figure 4, B and C), promoter regions close to 5′-UTR appear to have more TR occurrences in land plants. In particular, the UI200 (upstream intergenic 200 nt) regions display a strong positive correlation with 5′-UTR in terms of relative TR densities for land plants (*r* = 0.755; *P* = 1.998e−05).

### The nucleotide content of the most abundant TR motifs are influenced by GC content

As shown in Table S3, all 22 dicots have GC contents ranging from 32.40% to 39.56%, five monocots with GC contents ranging from 43.57% to 46.14%, and two green algae with GC contents ranging from 55.70% to 63.45%. For the two bryophyte species, the GC content of moss *Physcomitrella patens* (33.60%) is within the range of dicots, whereas spikemoss *Selaginella moellendorffii* (45.25%) is within monocots. GC contents in a genome-wide scale seem to show the following pattern: green algae > monocots > dicots. However, GC contents vary greatly among different intragenic and intergenic regions. In intragenic regions of dicots (Table 1 and Table S3), the highest and lowest GC contents are detected in CDS (44.34%) and introns (33.19%), respectively, and 5′-UTR has the second highest GC contents (39.89%). In intergenic regions of dicots, GC contents vary from 31.75% to 35.56%. In intragenic regions of monocots and bryophytes, the highest and lowest GC contents are detected in 5′-UTR (54.79%) and intron (39.15%), respectively, and CDS has the second highest content (53.32%). In intergenic regions of monocots and bryophytes, GC contents change from 42.39% to 49.20%. In green algae, the highest GC content is detected in CDS (66.17%) and lowest is in 5′-UTR (52.82%) and its adjacent intergenic region (UI200; 52.32%). The 3′-UTR and other intergenic regions in green algae have GC contents ranging from 53.75% to 57.38%. Different from both monocots and dicots, introns in green algae show the second highest GC content (58.38%).

**Table 1:** The most frequent TR motifs and GC contents in different genomic regions

Group Name (Genome GC Content Range)	Region	Average GC Content, %	Top Motifs
Dicots (32%–40%)	Whole genome	35.61	A/T; AT; ATT/AAT, AAG/CTT
	UI1000	32.63	A/T; AT; ATT/AAT, AAG/CTT
	UI500	31.75	A/T; AT; ATT/AAT
	UI200	35.56	A/T; AT, CT; CTT/AAG
	5′UTR	39.89	CT/AG; CTT/AAG
	CDS	44.34	AAG/CTT
	Intron	33.19	T; AT; ATT
	3′UTR	35. 41	T; AT; ATT/AAT
	DI200	34.58	T/A; AT, AG; AAT/ATT, AAG/CTT
	DI500	32.78	T/A; AT; AAT/ATT, AAG/CTT
	DI1000	33.50	T/A; AT; AAT/ATT, AAG/CTT
Monocots and bryophytes (34%–55%)	Whole genome	43.29	AT; AAT/ATT, CCG/CGG
	UI1000	42.39	AT; AAT/ATT, CCG/CGG
	UI500	43.38	AT; ATT, CCG
	UI200	49.20	AT; CCG
	5′UTR	54.79	AG/CT; CCG
	CDS	53.32	CGG/CCG
	Intron	39.15	C; CT
	3′UTR	42.34	CTT/AAG, CCG, GT
	DI200	45.27	AT; CGG/CCG
	DI500	42.67	AT; CGG/CCG
	DI1000	42.56	AT; CGG/CCG
Green algae (>55%)	Whole genome	59.58	AC/GT; CCG/CGG; AAGCATATGCGATCTGC
	UI1000	57.38	AC/GT; CCG/CGG; AAGCATATGCGATCTGC
	UI500	55.84	GT/AC
	UI200	52.32	GT/AC
	5′UTR	52.82	AGC
	CDS	66.17	CGG/CGG, AGC
	Intron	58.38	GT/AC
	3′UTR	55.42	GCT/AGC; GT
	DI200	53.75	GT/AC
	DI500	56.32	GT/AC; AAGCATATGCGATCTGC
	DI1000	57.16	GT/AC; AAGCATATGCGATCTGC

Our data suggest a clear relationship between GC contents and nucleotide content of the most frequent TR motifs detected within either a whole genome or individual intragenic or intergenic regions. If a high GC content is detected within a given region, then the abundant TR motifs will preferably be GC-rich. In dicots, the most abundant TRs have repeat unit sizes of mononucleotides, dinucleotides, and trinucleotides, except CDS in which trinucleotide TR motifs are the most frequent, and then tri-fold TR motifs (*e.g.*, hexanucleotide and nine-nucleotide motifs) are the second most abundant (Table 1). As shown in Table S4, the top TR motifs in dicots are dinucleotide motifs (16.87%, *e.g.*, AT), mononucleotide motifs (14.48%, *e.g.*, A/T), and trinucleotide motifs (9.17%, *e.g.*, ATT/AAT and AAG/CTT). Also, 4-bp to 7-bp motifs still show high frequencies (>3%), whereas other longer motifs have low frequencies (≤2%), except 39-nucleotide motifs (3.92%). This exception is caused by the dramatically high frequency of 39-nucleotide motifs detected in *R. communis* (69.57%) (Table S4). Moreover, only AT motifs (*e.g.*, T, AT, and ATT) are detected in introns in dicots where the lowest GC content is evident in comparison with other intragenic regions (Table 1). Different from dicots, mononucleotide motifs are lower in frequency (8.73%), whereas trinucleotide motifs (14.12%) and dinucleotide motifs (13.05%) are obviously preferred in monocots (>13%) (Table S4). Meanwhile, GC-rich motifs like CGG/GCC are more frequently found in monocots than in dicots because of their higher GC contents. Although trinucleotide and tri-fold nucleotide motifs are still dominant in CDS regions in monocots, those are essentially GC-rich motifs (*e.g.*, CGG/GCC). Interestingly, dinucleotide (16.69%) and 12-nucleotide (17.48%) motifs have higher frequencies in two bryophytes (Table S4), because dramatically high dinucleotide (26.79%) and 12-nucleotide (31.11%) motifs are detected in *P. patens* and *S. moellendorffii*, respectively. In green algae, mononucleotide TR motifs show an extremely low frequency (0.94% only) (Table S4). In green alga *C. reinhardtii*, dinucleotide GT/AC motifs are dominantly used in all intragenic and intergenic regions except 5′-UTR and CDS regions, where trinucleotide AGC and CGG/CGG motifs are frequently used. In green alga *V. carteri*, the long 17-nucleotide motifs are frequently found in all intragenic regions except CDS regions, where trinucleotide CGG and AGC are frequent. Interestingly, the top three frequent motifs in *V. carteri* are 17-nucleotide motifs (8.18%), trinucleotide motifs (7.44%), and 50-nucleotide motifs (7.39%) (Table S4).

Based on the analysis of 198 experimentally verified plant promoter sequences downloaded from EPD (Eukaryotic Promoter Database), the abundant TR motif units are mononucleotides, dinucleotides, and tetranucleotides, and the top-ranked frequent motifs are A-rich and AT-rich (*e.g.*, A/T, AT, ATGC, CTTT, and ATTT), similar to our results in the intergenic region adjacent to 5′-UTR (*i.e.*, UI200 regions) in the dicots and monocots.

### TR distribution and frequency profiles in intragenic (5′-UTR, CDS, intron, and 3′-UTR) and intergenic regions

**Figure 5:**
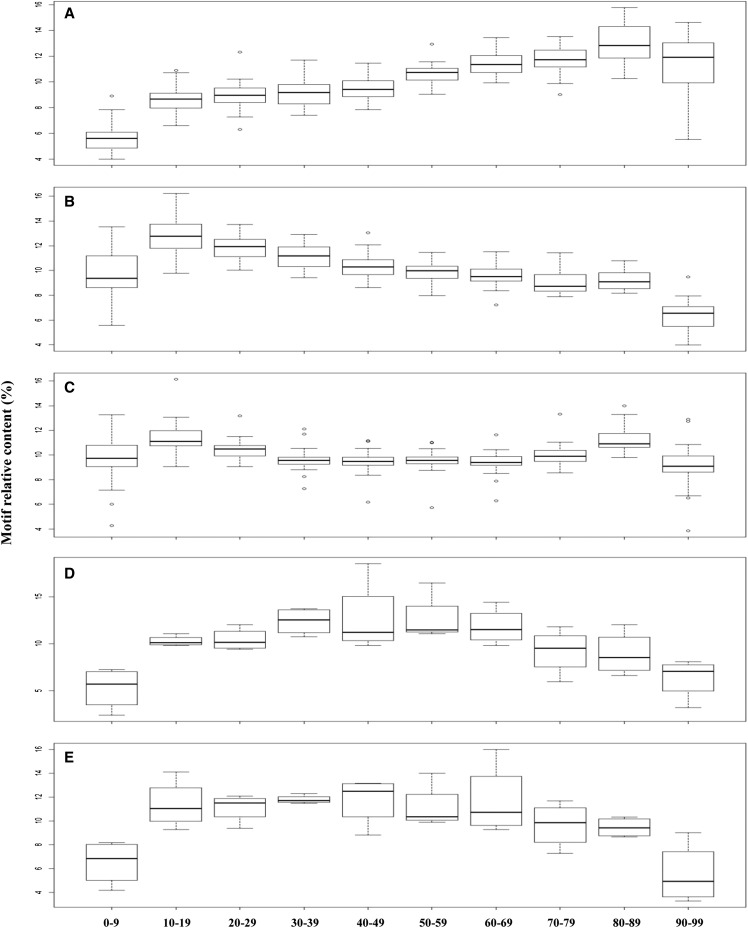
The relative distribution position of TRs in the intron and intergenic regions. (A) UI200 region in dicots and monocots. (B) DI200 region in dicots and monocots. (C) Intron region in the 31 investigated species. (D) UI200 region in bryophytes and green algae. (E) DI200 region in bryophytes and green algae.

As shown in Figure 5A and Table S5, within the upstream intergenic UI200 regions of both dicots and monocots, the highest and lowest relative TR motif contents are found in the 80–89 (*P* < 0.001, F-test from ANOVA; *P* < 0.01, HSD test) and 0–9 intervals (*P* < 0.001, F-test from ANOVA; *P* < 0.01, HSD test), respectively. This suggests that the distribution of TR motifs is significantly toward the 3′ ends of UI200, closer to 5′ ends of genes. In contrast, within the downstream intergenic DI200 regions (Figure 5B and Table S5), TR motifs are shown to distribute significantly toward the 5′ ends of DI200, closer to 3′ ends of genes, considering that the highest and lowest relative motif contents are detected in 10–19 (*P* < 0.001, F-test from ANOVA; *P* < 0.01, except compared with 20–29, where *P =* 0.13, HSD test) and 90–99 (*P* < 0.001, F-test from ANOVA; *P* < 0.01, HSD test) intervals, respectively. Interestingly, the progressively increasing trend of motif frequency toward gene ends does not keep in the subregions that are immediately adjacent to gene ends (*e.g.*, the interval 90–99 in Figure 5A and the interval 0–9 in Figure 5B). Meanwhile, TR motif frequencies appear to be relatively consistent within 5′-UTR, 3′-UTR, and CDS regions (Figure S1), except near their ends (*i.e.*, the interval 0–9 and the interval 90–99) where lower frequencies are detected. Within the introns of all 31 species, more motifs are significantly detected in 10–19 (*P* < 0.001, F-test from ANOVA; *P* < 0.05, HSD test) and 80–89 intervals (*P* < 0.001, F-test from ANOVA; *P* < 0.01, except compared with 20–29, where *P* = 0.19, HSD test) intervals. So, it is deduced that TR motifs are more frequently distributed toward intron ends forming a U-shape (Figure 5C), which has a different trend than intergenic UI200 and DI200 regions.

Different from dicots and monocots, bryophytes and green algae show special trends in TR distribution profiles in both intergenic UI200 and DI200 regions: more motifs are detected in the middle intervals (Figure 5, D and E and Table S5) and no progressive increase or decrease trend is observed. However, the TR distribution profiles within intragenic regions are similar to dicots and monocots (Figure S1 and Table S5).

**Figure 6:**
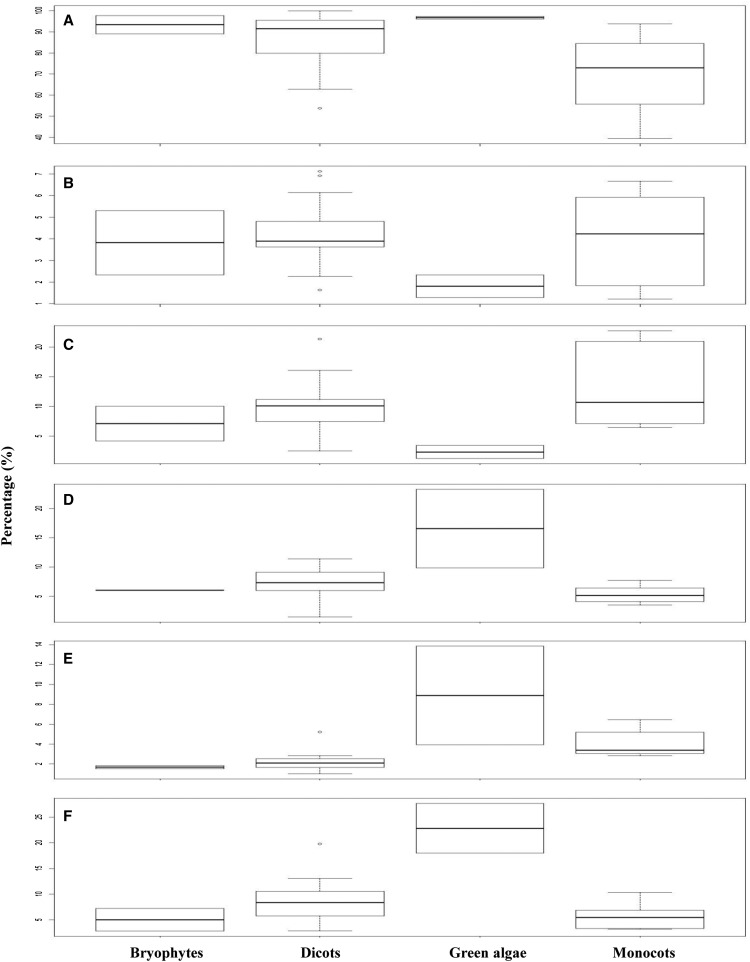
The percentage of TRs in different intragenic and intergenic regions. (A) mRNAs. (B) UI200 region. (C) 5′-UTR region. (D) 3′-UTR region. (E) CDS region. (F) Intron region.

As shown in Figure 6 and Table S6, we have determined TR occurrences among all annotated genes, their intragenic regions, and adjacent promoter regions for four groups of all 31 species (bryophytes, monocots, dicots, and green algae). First, ∼84% of all annotated mRNAs (or genes; some genes have more than one mRNA annotated) possess TRs (Figure 6A and Table S6), and no significant difference is detected among four groups by F-test or HSD test. Interestingly, monocots show less TR frequency than the other three groups, but the difference is not statistically significant. In UI200 and 5′-UTR regions (Figure 6, B and C), ∼4% and 10% of the annotated mRNAs have TRs, respectively, except for green algae, in which only ∼2% are found with TRs in both regions. The difference in TR frequencies in UI200 and 5′-UTR regions of all annotated mRNAs is not significant among the four groups. However, as shown in Figure 6 D, E, and F, green algae display significantly higher TR frequencies in 3′-UTR (∼17%), CDS (∼9%), and intron (∼23%) regions for all annotated mRNAs in comparison with the other three groups: bryophytes, monocots, and dicots (*P* < 0.01, F-test from ANOVA; *P* < 0.01, HSD test).

Utilizing GOEAST (Gene Ontology Enrichment Analysis Software Toolkit) (Zheng and Wang 2008), the GO terms of *C. reinhardtii* genes with TRs in introns were analyzed. As shown in Table 2, the most highly enriched GO terms involve catalytic activity (*P* = 5.384e−35) and hydrolase activity (*P* = 1.344e−10). In green alga *V. carteri*, the most significant GO functions mainly involve ribosomal proteins and heat shock proteins in the genes with TRs in introns (Table S7). Such results suggest that the TRs could involve RNA and/or protein activity in intron processing.

**Table 2:** The most significant GO functions of genes with TRs in introns in *C. reinhardtii*

Term	*P*
Catalytic activity	5.384e−35
Hydrolase activity	1.344e−10
Oxidoreductase activity	7.792e−6
Peptidase activity	1.109e−4
Peptidase activity acting on L-amino acid peptides	1.766e−4
Endopeptidase activity	5.793e−4

## Discussion

### The variation of TR densities in different genomes

In a genome-wide study of TRs using 12 species including two fungi (*S. cerevisiae* and *Neurospora crassa*), one green alga (*Ostreococcus lucimarinus*), one plant (*A. thalina*), three vertebrates (*Homo sapiens*, *Mus musculus*, *Gallus gallus*), one nematode (*Caenorhabditis elegans*), and three arthropods (*Daphnia pulex*, *Drosophila melanogaster*, *Apis mellifera*), Mayer *et al.* (2010) detected weak, but not significant, correlation between the genome sizes and TR densities (*r* = 0.483; *P* = 0.111). In three plant families Brassicaceae, Solanaceae, and Poaceae (da Maia *et al.* 2009), the association between genome sizes and TR densities detected in mRNA/cDNA data was also not found. In a recent study of 257 virus genomes, the relative SSR densities (*i.e.*, SSRs sequence base pairs per kilo genomic base pairs) showed quite weak correlation with genome size (Zhao *et al.* 2012). Our analysis showed no significant relationship detected between TR density and genome size in green algae and plants (Figure 3). Furthermore, it was obviously shown that TR densities have species-specific features rather than group-based features, like the two green algae; such results coincided with the SSR density variation detected in 25 algae and plants (von Stackelberg *et al.* 2006). There was a weak positive, but not significant, correlation detected between genome sizes and TR densities for both compact genomes (like viruses) and genomes with lots of intergenic regions (like plants), suggesting that TRs might have not contributed significantly to the genome size expansion in evolution.

### The variation of TR densities in different intragenic and intergenic regions

In Arabidopsis and rice, TRs are significantly enriched within 5′-UTRs (Fujimori *et al.* 2003; Zhang *et al.* 2004; Lawson and Zhang 2006). In our study, both dicot and monocot plants possess the first and second highest TR densities in 5′-UTRs and their immediate upstream intergenic regions (*i.e.*, UI200), which belong to the promoter regions where core promoter elements are often represented (Kokulapalan 2011) (Figure 4, B and C). The 5′-UTRs are thought to be the hot spots for TRs in eukaryotes. Previous studies of genes for light and salicylic acid responses (Li *et al.* 2004; Zhang *et al.* 2006) suggested that TRs in 5′-UTRs might be involved in the transcription and/or translation regulation. It has been reported that as many as 25% genes in yeast *S. cerevisiae* have TRs in the promoter regions (Vinces *et al.* 2009). Our study also demonstrated that ∼4–25% of genes in dicots and monocots possess TR in both 5′-UTR and promoter UI200 regions (Figure 6, B and C). In both dicots and monocots, TR abundance is the least in the CDS region, indicating that low TR abundance may decrease the evolvability of proteins. This is reasonable because it has been demonstrated that the mutations of CDS could cause protein functional changes, loss of function, and protein truncation (Li *et al.* 2004). Interestingly, intron and 3′-UTR regions have much lower TR densities in monocots than in dicots. Such TR differences between dicots and monocots are still not clear in their biological meanings.

In the two green algae we examined, the first and second highest TR densities were detected in intron and CDS regions, respectively (Figure 4A), which was completely different from all other land plants. Our data show that green algae have significantly more intron sequences (32.85% and 37.03% in the whole genome in *C. reinhardtii* and *V. carteri*) compared with land plants (average, 15.73% ). This may imply that in green algae the TRs in the intron and CDS regions are not randomly expanded and could be involved in intron-related or CDS-related activities and in RNA processing (*e.g.*, exon splicing). In fact, our GO analysis for *C. reinhardtii* genes with TRs in intons showed that the most significant GO functions were catalytic activity and hydrolase activity (Table 2). Those functions indicate that the genes with rich TR motifs in their introns could be involved in protein synthesis and degradation.

### The top TR motifs are influenced by GC content

In our study, the top-ranked TR motifs are CT/AG and CTT/AAG in 5′-UTR in dicots. This is consistent with the results of Zhang *et al.* (2006) in which the motifs (CT/AG and CTT/AAG) were preferred in 5′-UTR in Arabidopsis and acted as regulatory elements for genes involved in light and salicylic acid responses (Zhang *et al.* 2006). Our results also showed that CDS regions are preferentially associated with trinucleotides and hexanucleotides motifs, which has been reported previously by other researchers (Subramanian *et al.* 2003; Fujimori *et al.* 2003; Li *et al.* 2004; Zhang *et al.* 2006; Mayer *et al.* 2010). It is suggested that there is strong evolutionary pressure against TR expansion in CDS than in introns to keep stable protein products (Dokholyan *et al.* 2000). Such a feature can help explain why tri-fold nucleotide motifs (*e.g.*, trinucleotide and hexanucleotide motifs) are more frequent than others to reduce potential translational frame shifting. Two green algae have the highest TR densities in introns and the relevant abundant motifs are dinucleotide GT/AC in our study. Canonical splicing signals GT and AG are located at the 5′ and 3′ ends of the intron, respectively. The abundant GT/AC dinucleotide TRs in introns might suggest that such repeats may be involved in exon splicing or alternative splicing in green algae (Gemayel *et al.* 2012).

In dicots, most TR motifs contain A and/or T nucleotide(s), whereas both A/T-rich motifs and CCG/CGG motifs are often used in monocots. However, A/T-rich motifs are rarely detected in the two green algae. Therefore, it is clear that the top TR motifs have a strong relationship with the GC content (Table 1). If there is high GC content, then the most frequent TR motifs prefer to be GC-rich instead of AT-rich. A similar relationship also has been demonstrated in 11 species (including green algae, bryophytes, ferns, gymnosperms, and angiosperms) (Victoria *et al.* 2011) and amino acid repeats in 10 angiosperms (Zhou *et al.* 2011).

In terms of repeat unit size length distribution (Table S4), mononucleotide motifs are not the most frequent TR motifs in all 31 investigated species. It is known that longer repeats (>6 bp) have high densities in *D. pulex* (Mayer *et al.* 2010). In our study, some longer repeats also show higher frequencies than many short TRs: 39-nucleotide motifs in dicot *R. communis*, 17-nucleotide and 50-nucleotide motifs in green alga *V. carteri*, and 12-nucleotide motifs in bryophyte *S. moellendorffii*. Therefore, this suggests that TRs are not generated randomly in genomes and longer TRs may play some roles in gene expression and regulation.

### The distribution and frequency of TRs in intragenic and intergenic regions

It is clear that the distribution of TR motifs in intergenic regions is significantly biased toward both the 5′ and 3′ ends of genes in dicots and monocots (Figure 5, A and B). TRs have been shown to locate within genes and regulatory regions and participate in transcriptional and translational regulation (Streelman and Kocher 2002; Rockman and Wray 2002; Li *et al.* 2002; Martin *et al.* 2005; Legendre *et al.* 2007; Vinces *et al.* 2009). In our study, the biased TR motif distribution in intergenic regions further supports this notion.

In introns of all 31 species, especially in the two green algae, more abundant TR motifs are significantly detected toward the ends of introns. Interestingly, SSR densities in CDS regions of 42 prokayrote genomes also show a similar U-shape profile (Lin and Kussell 2011). Because introns contain important regulatory motifs for many biological processes, including splicing (Matlin *et al.* 2005; Barbazuk *et al.* 2008), our results suggest that the TRs in introns might have localization preference in their regulatory roles. Considering exon splicing that utilizes the canonical splicing signals (GT and AG) at the 5′ and 3′ end of introns and the GO functions of genes with TRs in introns (Table 2), we believe that the highly abundant TRs in introns, especially in the two green algae, may involve with both constitutive and alternative splicing activities.

The frequencies of TRs are consistent with the TR density variations in the four different groups. It has been shown that 5′-UTR and UI200 have much higher TR densities in dicots, monocots, and bryophytes, whereas higher TR densities are found in intron and CDS regions in green algae (Figure 4 and Figure 6). Comparatively speaking, there are more TRs (densities and frequencies) in 5′-UTR and promoter (UI200) regions in land plants (dicots, monocots, and bryophytes), whereas green algae have more TRs in intron and CDS regions.

In this study, the genome assemblies and gene annotations were obtained from Phytozome version 8.0. Within this release, some species (*e.g.*, Arabidopsis and rice) apparently have better, high-quality gene annotations than other species (*e.g.*, papaya and apple without UTR annotation). We also noticed that many genome assemblies have unfinished gaps (*e.g.*, …NNN…). Perhaps this is attributable to the highly repetitive nature of the sequences and the limitation of current sequencing technologies. On other hand, our data analysis is obviously biased toward dicot plants because the species number available in Phytozome version 8.0 is not balanced for all four groups: two species in green algae, two in bryophytes, five in monocots, and 22 in dicots. Another limitation in our data analysis is the repeat unit size selection. Ideally, we should have examined all TRs with the repeat unit size of 1–100 (*i.e.*, covering all microsatellites and minisatellites) or even longer. Unfortunately, we decided to examine the TR motifs of 1–50 bp because of the constraints in both current bioinformatics tools and the demanding computational resources required for processing all 31 genomes (*i.e.*, CPU, memory, and execution periods). Clearly, these limitations will affect the quality of our data analysis results presented in this article to some extent. With the rapid advances in sequencing and computational technologies and with the rapid increase of transcriptomics data, we can expect more high-quality, accurate genome assemblies and gene annotations available for in-depth TR analyses involving more plant and green algal species. This will definitely help us improve our understanding of the evolution of TRs and their roles in gene expression regulation.

## Conclusions

It is known that TRs involve plenty of roles in gene expression and genome evolution. In this study, as the first systematic examination of TRs in plant and green alga genomes, we found that TR density has no significantly discernible relationship with genome size, and TRs display nonrandom distribution within both intragenic and intergenic regions, suggesting that they might have been involved in transcriptional or translational regulation in plants and green algae. Obviously, more research work is needed to facilitate our understanding of TRs in terms of their motif features and potential biological functions, as well as their evolutionary trends in land plants and green algae.

## Supplementary Material

Supporting Information
