# Supplementary material for: Genome-Wide Analysis of Tandem Repeats in Plants and Green Algae
Source: G3 (Bethesda). 2013 Nov 5;4(1):67–78. doi: 10.1534/g3.113.008524 (PMC3887541; doi:10.1534/g3.113.008524)
Supplement: Supporting Information [file supp_4_1_67__index.html]

Genome-Wide Analysis of Tandem Repeats in Plants and Green Algae — Supporting Information 

# Genome-Wide Analysis of Tandem Repeats in Plants and Green Algae

## Supporting Information for Zhao *et al.*, 2014

**Files in this Data Supplement:**

- Supporting Information - Figure S1 and Tables S1-S7 (PDF, 299 KB)
- Figure S1 - The relative distribution position of TRs in the 3 intragenic regions. (PDF, 235 KB)
- Table S1 - The relative TR densities in the 31 investigated species (.xlsx, 10 KB)
- Table S2 - The means and SD values of relative TR densities shown in Figures 4-6 (.xlsx, 16 KB)
- Table S3 - GC contents and top frequent TR motifs in the 31 investigated species (.xlsx, 17 KB)
- Table S4 - 1-50 bp TR motif length distribution (%) in all 31 investigated species (.xlsx, 49 KB)
- Table S5 - TR distribution in intragenic and intergenic regions in the 31 investigated species (.xlsx, 496 KB)
- Table S6 - The TR numbers and percentages in intragenic and intergenic regions in the 31 investigated species (.xlsx, 15 KB)
- Table S7 - The list of GO functions from the genes with TRs in intron regions in green alga *V. carteri* (.xlsx, 945 KB)
