# Supplementary material for: Genome-Wide Analysis of Tandem Repeats in Plants and Green Algae
Source: G3 (Bethesda). 2013 Nov 5;4(1):67–78. doi: 10.1534/g3.113.008524 (PMC3887541; doi:10.1534/g3.113.008524)
Supplement: Supporting Information [file supp_g3.113.008524_008524SI.pdf]

G3 Genes | Genomes | Genetics

Genome-wide analysis of tandem repeats in plants and green algae

Zhixin Zhao[1], Cheng Guo[1], Sreeskandarajan Sutharzan[1], Pei Li[1,2], Craig S Echt[3], Jie Zhang[4] and Chun Liang[1*]

1 Department of Botany, Miami University, Oxford, Ohio 45056, USA
2 Department of Automation, Xiamen University, Xiamen, 361005, China
3 Southern Research Station, USDA Forest Service, Saucier, MS 39574, USA
4 State Key Laboratory for Biology of Plant Diseases and Insect Pests, Institute of Plant Protection, Chinese Academy of Agricultural Science, Beijing 100193, China

*Corresponding author

Email addresses:
ZZ: zhaoz@miamioh.edu
CG: guoc2@miamioh.edu
SS: sreesks@miamioh.edu
PL: peil@miamioh.edu
CSE: cecht@fs.fed.us
JZ: zhangjie05@caas.cn
CL: liangc@miamioh.edu

DOI: 10.1534/g3.113.008524

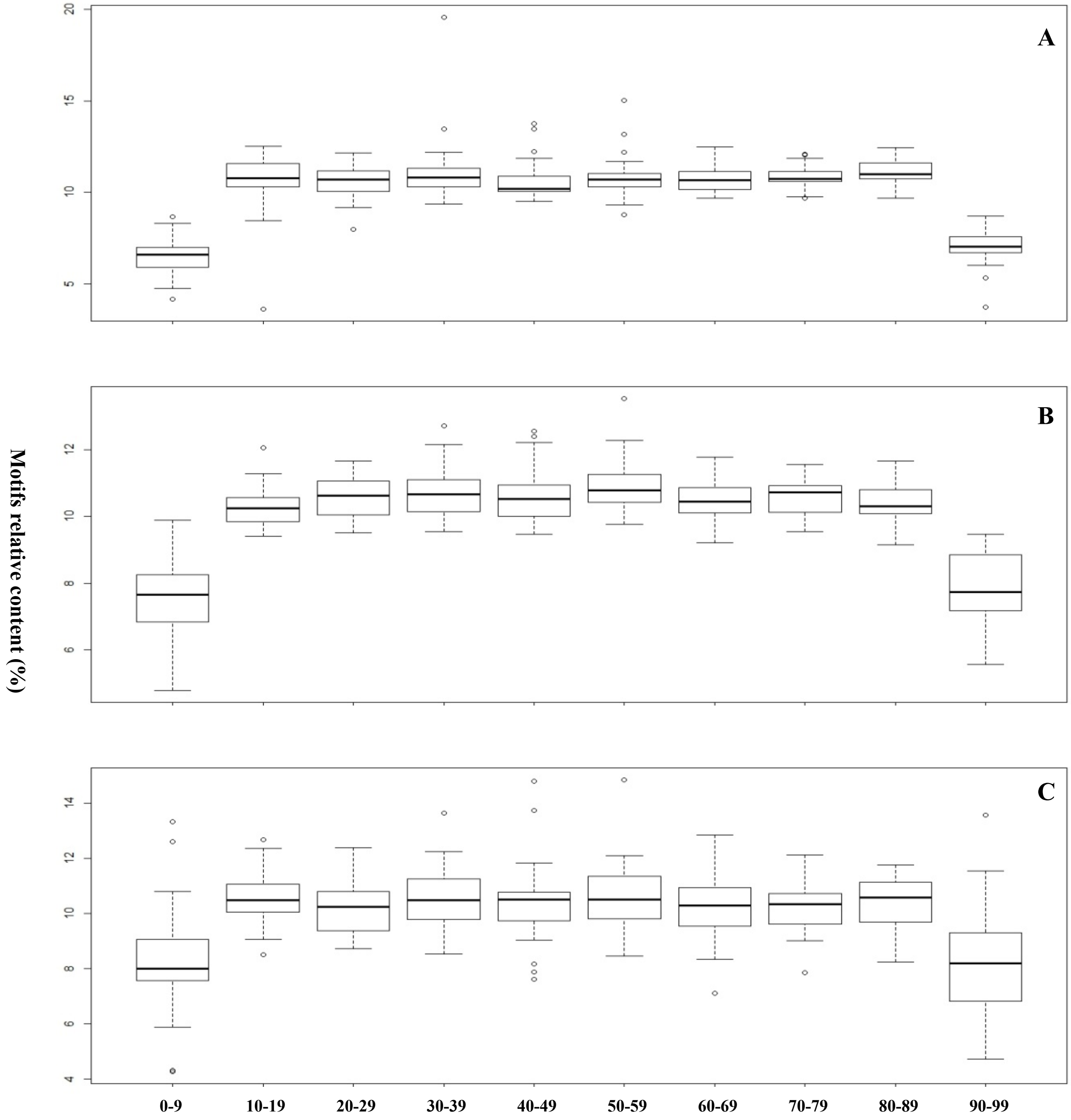

Figure S1 The relative distribution position of TRs in the 3 intragenic regions. (A) 5'-UTR regions in the 27 investigated species; (B) CDS regions in the 31 investigated species; (C) 3'-UTR regions in the 27 investigated species.

Tables S1-S7 are available for download at http://www.g3journal.org/lookup/suppl/doi:10.1534/g3.113.008524/-/DC1.

Table S1 The relative TR densities in the 31 investigated species.

Table S2 The means and SD values of relative TR densities shown in Figures 4-6.

Table S3 GC contents and top frequent TR motifs in the 31 investigated species.

Table S4 1-50 bp TR motif length distribution (%) in all 31 investigated species.

Table S5 TR distribution in intragenic and intergenic regions in the 31 investigated species.

Table S6 The TR numbers and percentages in intragenic and intergenic regions in the 31 investigated species.

Table S7 The list of GO functions from the genes with TRs in intron regions in green alga *V.carteri*.
