# Supplementary material for: Genome-Wide Analysis of Tandem Repeats in Plants and Green Algae
Source: G3 (Bethesda). 2013 Nov 5;4(1):67–78. doi: 10.1534/g3.113.008524 (PMC3887541; doi:10.1534/g3.113.008524)
Supplement: Supporting Information [file supp_g3.113.008524_FigureS1.pdf]

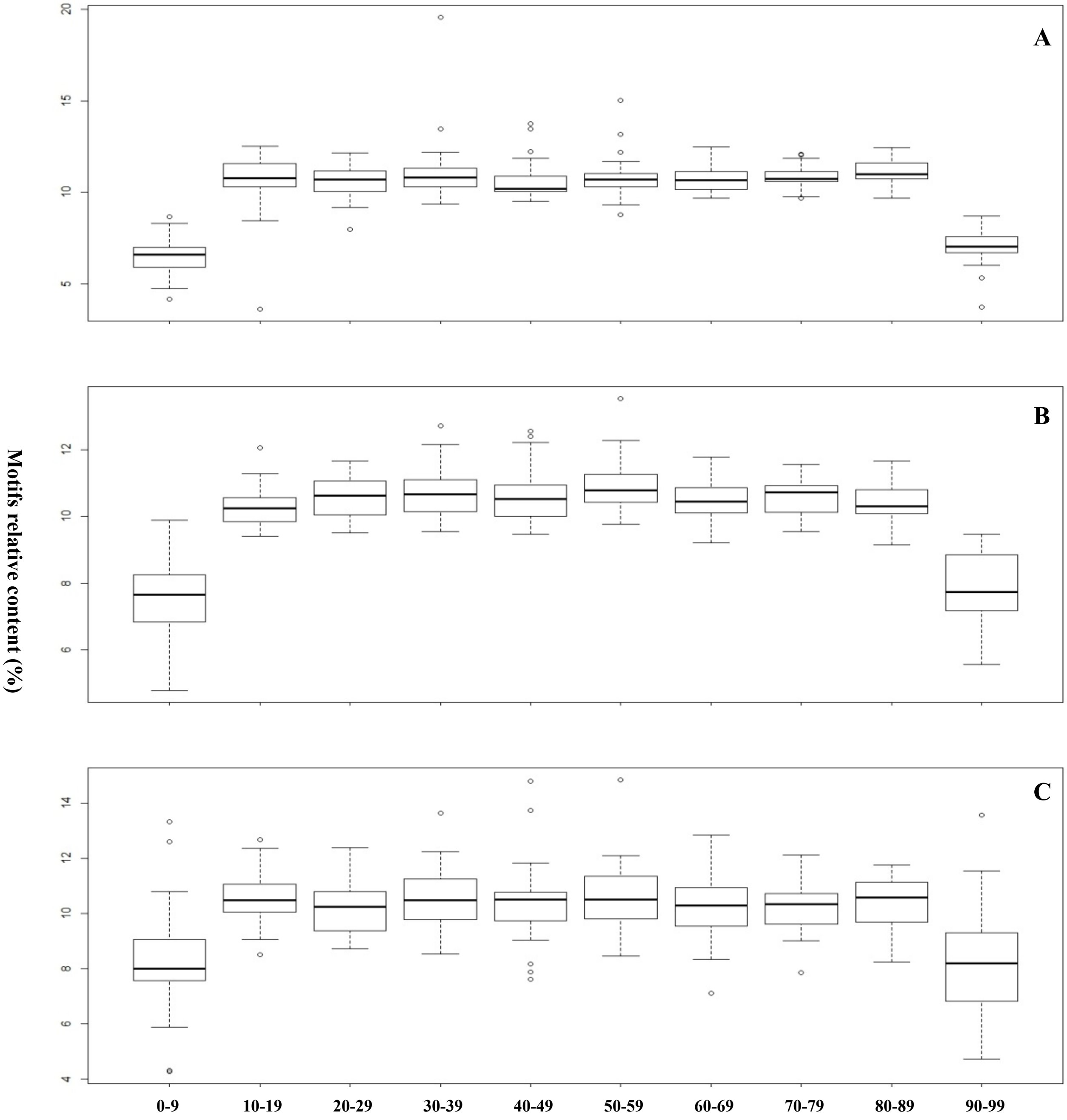

Figure S1 The relative distribution position of TRs in the 3 intragenic regions. (A) 5'-UTR regions in the 27 investigated species; (B) CDS regions in the 31 investigated species; (C) 3'-UTR regions in the 27 investigated species.
